# An Evaluation of the Biocompatibility and Chemical Properties of Two Bioceramic Root Canal Sealers in a Sealer Extrusion Model of Rat Molars

**DOI:** 10.3390/jfb16010014

**Published:** 2025-01-04

**Authors:** Shintaro Takahara, Naoki Edanami, Razi Saifullah Ibn Belal, Kunihiko Yoshiba, Shoji Takenaka, Naoto Ohkura, Nagako Yoshiba, Susan Gomez-Kasimoto, Yuichiro Noiri

**Affiliations:** 1 Division of Cariology, Operative Dentistry and Endodontics, Department of Oral Health Science, Niigata University Graduate School of Medical and Dental Sciences, Niigata 951-8126, Japan; takahara@dent.niigata-u.ac.jp (S.T.); dr.razi3187@gmail.com (R.S.I.B.); stakenaka@dent.niigata-u.ac.jp (S.T.); ohkura@dent.niigata-u.ac.jp (N.O.); susangk@dent.niigata-u.ac.jp (S.G.-K.); noiri@dent.niigata-u.ac.jp (Y.N.); 2 Division of Oral Science for Health Promotion, Department of Oral Health and Welfare, Niigata University Graduate School of Medical and Dental Sciences, Niigata 951-8126, Japan; yoshiba@dent.niigata-u.ac.jp (K.Y.); nagako@dent.niigata-u.ac.jp (N.Y.)

**Keywords:** bioceramic root canal sealer, sealer extrusion, biocompatibility, silicon element release, hydroxyapatite formation

## Abstract

This study assessed the biocompatibility and chemical properties of two bioceramic root canal sealers, EndoSequence BC Sealer (EBC) and Nishika Canal Sealer BG (NBG), using a sealer extrusion model. Eight-week-old male Wistar rats were used. The mesial root canals of the upper first molars were pulpectomized and overfilled with EBC, NBG, or, as reference, epoxy resin-based AH Plus (AHP). After 28 days, periapical tissue reactions were assessed using microcomputed tomography and histological staining. The elemental composition and chemical composition of the extruded EBC and NBG were analyzed at Day 1 and 28 using an electron probe microanalyzer and micro-Raman spectroscopy. No periapical lesions were observed with the sealer extrusion. Additionally, inflammation around the extruded EBC and NBG was minimal to mild on Day 28, whereas moderate inflammation was found around the extruded AHP. Silicon concentration in the extruded EBC and NBG decreased significantly from Day 1 to 28, with almost no silicon present on Day 28. Furthermore, the extruded EBC and NBG became calcium- and phosphorus-rich, showing a Raman band for hydroxyapatite on Day 28. In conclusion, EBC and NBG demonstrated favorable biocompatibility and the ability to release silicon elements and produce hydroxyapatite when extruded into the periapical tissues. AHP showed moderate periapical tissue irritancy.

## 1. Introduction

Root canal obturation is a crucial step in nonsurgical root canal treatment following the cleaning and shaping of the root canal. The use of a root canal sealer in combination with core filling materials, such as gutta-percha points, is essential to achieve a bacteria- and fluid-proof seal in the root canal system. The absence of a root canal sealer compromises the filling’s integrity, leading to increased leakage over time and ultimately causing endodontic treatment failure [1].

Advancements in material science have resulted in the development of various types of root canal sealers, each with unique physicochemical and biological properties. These include sealers based on zinc oxide–eugenol, zinc oxide–fatty acid, epoxy resin, methacrylate resin, salicylate, glass ionomers, silicone, and bioceramics [2]. While zinc oxide–eugenol and epoxy resin-based sealers have been widely used for many years because of their satisfactory physicochemical properties [3], bioceramic sealers have recently gained popularity for their ability to not only seal the root canal system but also release bioactive ions that can modulate inflammation [4] and promote periapical tissue healing [5].

EndoSequence BC Sealer (EBC; Brasseler, Savannah, GA, USA), also known as iRoot SP and TotalFill BC, is one of the most frequently investigated bioceramic sealers containing tricalcium silicate. It is available in a ready-to-use injectable paste form, making it easy to apply in root canals. Additionally, it hardens in a moist environment through a hydration reaction of the tri- and dicalcium silicate components, forming calcium silicate hydrate and slightly expanding during the hardening process, which is advantageous for root canal sealing [6]. Previous studies have confirmed its good physicochemical properties [7] and excellent sealing performance [8]. Furthermore, EBC extract media have been shown to promote the mineralization of human periodontal ligament stem cells [9] and murine osteoblast precursor cells [10].

Nishika Canal Sealer BG (NBG; Nippon Shika Yakuhin, Yamaguchi, Japan) is a recently developed bioceramic sealer containing bioactive glass. NBG has a paste–paste formulation, and the mixed paste hardens through a chelation reaction between magnesium oxide and fatty acids. Bioactive glass serves as a filler particle in NBG. The sealing ability of NBG has been found to be comparable to that of EBC and superior to that of a zinc oxide–eugenol sealer in a dye leakage test [11]. Additionally, NBG releases more silicate ions than tricalcium silicate-containing bioceramic sealers and more effectively stimulates the osteogenesis of human periodontal ligament stem cells and the angiogenesis of human umbilical vein endothelial cells [12].

Biocompatibility refers to the ability to interact with a living system without causing any adverse effects [13]. It is crucial to investigate the biocompatibility of newly developed root canal sealers, as they may come into direct contact with periapical tissues in cases of accidental sealer extrusion. Previous studies have reported that EBC and NBG caused minimal inflammation when implanted in the subcutaneous tissue of rats [14,15]. Furthermore, EBC did not hinder bone tissue repair when implanted in surgical cavities in rat femurs [16]. However, different cell types and tissues may respond differently to foreign substances, raising concerns about the clinical relevance of these findings based on subcutaneous tissue and femur models. To date, no studies have histologically examined the responses of periapical tissues to the extrusion of EBC, NBG, or other bioceramic sealers, except for a single case report involving EBC [17].

Therefore, the present study aimed to assess the biocompatibility of EBC and NBG through intentional sealer extrusion in rat molars. An epoxy resin-based sealer, AH Plus (AHP; Dentsply Sirona, York, PA, USA), was used as a reference, because it is widely considered the gold standard among root canal sealers owing to its excellent mechanical properties [2]. To assess the biocompatibility of the sealers, we quantified the number of inflammatory cells and interleukin-1 beta (IL-1β)-immunopositive cells surrounding the extruded sealers. Additionally, the present study examined the compositional changes of the extruded EBC and NBG to better understand their chemical properties. These changes were analyzed using electron probe microanalysis (EPMA) and micro-Raman spectroscopy. EPMA facilitates highly precise quantitative analysis of elemental distribution and concentration with excellent spatial resolution [18], while micro-Raman spectroscopy enables nondestructive characterization of molecular structures, chemical bonds, and phase transitions [19].

The first null hypothesis of the present study was that EBC and NBG do not exhibit biocompatibility in periapical tissues. The second null hypothesis was that EBC and NBG do not undergo compositional changes in periapical tissues.

## 2. Materials and Methods

### 2.1. Materials

Two bioceramic sealers, tricalcium silicate-containing EBC and bioactive glass-containing NBG, were assessed. AHP served as a reference sealer. The compositions of these sealers are presented in Table 1.

### 2.2. Ethical Approval

All animal experiments were conducted in accordance with the guidelines of the Committee on Animal Experimentation of Niigata University (approval number: SA01256) and complied with all international, national, and institutional guidelines for the care and use of animals.

### 2.3. Animals

**Figure 1:**
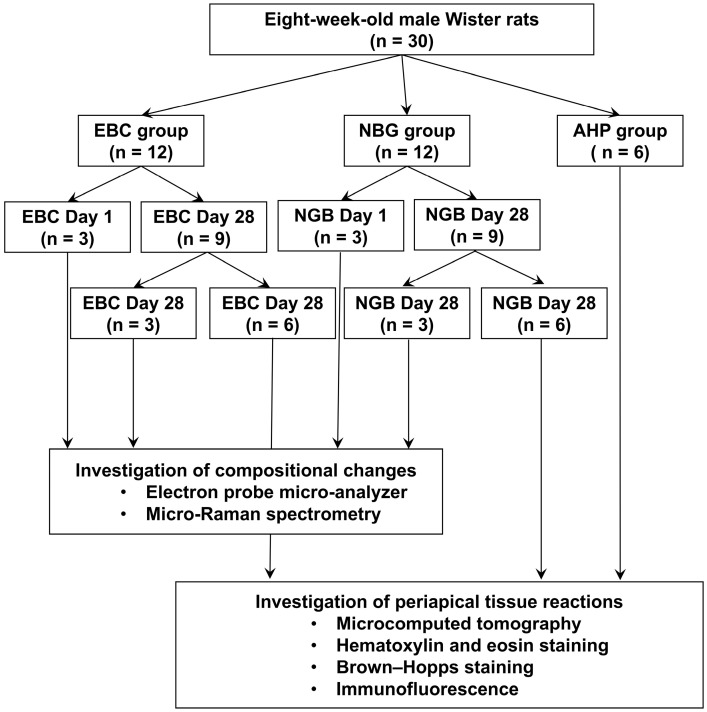
Experimental flowchart.

Eight-week-old male Wistar rats (n = 30) weighing 240 to 260 g were purchased from Clea Japan (Tokyo, Japan). The rats were housed under standardized conditions (temperature: 23 °C ± 2 °C; humidity: 40–70%; 12 h light/dark cycle) with ad libitum access to water and a commercial pellet diet. The upper left first molars of the rats were treated to assess the biocompatibility and chemical properties of EBC and NBG, whereas the upper right first molars served as untreated controls. Figure 1 shows a flowchart of the experiments.

### 2.4. Treatments

All surgical procedures were conducted using a 20× microscope field of view (S9D microscope; Leica, Wetzlar, Germany). The animals were anesthetized with an intraperitoneal administration of medetomidine hydrochloride, midazolam, and butorphanol (Fujifilm Wako, Osaka, Japan). The surgical site was disinfected by swabbing with a cotton ball soaked in a 10% povidone-iodine solution. The pulp chamber of the upper left first molars was accessed using an International Organization for Standardization 008 round carbide bur (Dentsply, Tulsa, OK, USA) attached to a low-speed handpiece, and the coronal pulp tissue was removed with the same round carbide bur. Subsequently, the radicular pulp tissue in the mesial root of the upper left first molars was removed using nickel–titanium rotary files (Race Evo; FKG, La Chaux-de-Fonds, Switzerland) in sizes #15 taper 0.04, #25 taper 0.04, and #30 taper 0.04 in a sequential manner. The working length for pulp removal was set at 3.5 mm based on the average root length of the teeth. The instrumented root canals were then irrigated with 2.5% sodium hypochlorite, followed by sterile saline, using 30-gauge needles (Navi tip; Ultradent, South Jordan, UT, USA). The canals were then dried with #30 sterile paper points (J Morita, Tokyo, Japan) and overfilled with EBC, NBG, or AHP. The overfilling procedure was as follows: A sealer was injected into the root canal using the sealer injection system (BG FIL; Nippon Shika Yakuhin). Hydraulic temporary cement (Caviton; GC, Tokyo, Japan) was placed at the root canal orifice, and a plugger (BL Kondenser; B&L Biotech, Anshan, Republic of Korea) was used to compress the Caviton cement by approximately 1 mm, allowing the sealer to extrude into the periapical tissues. The pulp chamber was then filled with Caviton, and the access opening was sealed with a bonding system (Clearfil Universal Bond Quick; Kuraray, Tokyo, Japan) and a flowable composite resin (Beautifil Flow; Shofu, Kyoto, Japan). The lower first molars were extracted to prevent fracture of the upper first molars. The animals were euthanized by carbon dioxide inhalation after Day 1 (*n* = 6) and 28 (*n* = 24) post-sealer extrusion, and the maxilla, containing the treated upper left first molars and the untreated upper right first molars, was harvested.

### 2.5. Periapical Tissue Reactions

#### 2.5.1. Specimens

Specimens retrieved on the 28th postoperative day (n = 6 for each material) were examined. The specimens were fixed in 4% paraformaldehyde for 24 h and then stored in phosphate-buffered saline.

#### 2.5.2. Microcomputed Tomography (CT)

The fixed specimens underwent scanning using a high-resolution micro-CT scanner (CosmoScan GX; Rigaku, Tokyo, Japan). The X-ray energy was set at 90 kV and 88 µA, with an exposure time of two minutes. The field of view was 5  ×  5  ×  5 mm, and the image resolution was 10 μm. Three-dimensional reconstruction images were generated using CosmoScan GX software (Rigaku). The presence or absence of a periapical lesion was assessed based on the periapical index developed by Estrela et al. [20]. A periapical lesion was considered present if a periapical radiolucency with a diameter exceeding 0.5 mm was observed.

#### 2.5.3. Hematoxylin and Eosin Staining

After the micro-CT scans, the specimens were demineralized in an ethylenediaminetetraacetic acid solution (Osteosoft; Merck, Darmstadt, Germany) for four weeks, dehydrated in ethanol, cleared in xylene, and embedded in paraffin blocks (Paraplast Plus; Sigma-Aldrich, St. Louis, MO, USA). The paraffin-embedded specimens were vertically sectioned along the mesial-to-distal axis of the upper first molars at a thickness of 5 μm using a microtome (HistoCore Multicut, Leica). The representative sections that intersected the central part of the extruded sealers (treated teeth) or normal apical foramen (untreated teeth) were stained with hematoxylin (Mayer’s Hematoxylin Solution; Fujifilm Wako) and eosin (1% Eosin Y Solution; Fujifilm Wako) and examined under a light microscope (Eclipse E800; Nikon, Tokyo, Japan). Inflammatory cells (neutrophils, lymphocytes, plasma cells, and macrophages) surrounding the extruded sealers were quantified under a 400× field of view (220 × 165 μm^2^) in three different areas per section, and the average count was determined. The identification of inflammatory cells was based on their morphological characteristics: neutrophils with multilobed nuclei; lymphocytes as rounded cells with a dense, round nucleus; plasma cells as large or ovoid cells with a round nucleus eccentrically positioned in their cytoplasm; and macrophages as irregularly shaped cells with an eccentric, indented nucleus [21]. The intensity of the inflammatory infiltrate was graded as absent/very few (<3 cells on average), mild (3–10 cells on average), moderate (10–25 cells on average), or severe (>25 cells on average) following the criteria of Holland et al. [22], with slight modifications. The histological analysis was performed by an observer blinded to the treatment groups.

#### 2.5.4. Brown–Hopps Staining

Brown–Hopps staining was applied using a staining kit (Muto Pure Chemicals, Tokyo, Japan) to investigate bacterial infection of the periapical tissues.

#### 2.5.5. Immunofluorescence

Immunofluorescence staining for IL-1β was performed as described in our previous study [23]. The primary antibody used was a rabbit anti-IL-1β polyclonal antibody (ab9722; Abcam, Cambridge, MA, USA), whereas the secondary antibody was Alexa Fluor 488-conjugated anti-rabbit IgG goat polyclonal antibody (Invitrogen, Carlsbad, CA, USA). For mounting, ProLong Diamond with DAPI (Thermo Fisher Scientific, Austin, TX, USA) was employed. IL-1β-positive cell density in the periapical connective tissue was quantified based on immunostained sections. Regions within 100 μm of the extruded sealers (treated teeth) and regions enclosed by a line perpendicular to the root apex and the periapical bone (untreated teeth) were analyzed. ImageJ software (version 1.53t; National Institutes of Health, Bethesda, MD, USA) was used to delineate the regions of interest and calculate their areas. The density of IL-1β-positive cells was determined by dividing the number of positive cells by the corresponding area. Statistical analyses were performed using Tukey’s test to compare differences between the groups, with the significance threshold being set at *p* < 0.05. All statistical analyses were conducted using the Statistical Package for the Social Sciences, version 10, for Windows (SPSS 10; SPSS Japan, Tokyo, Japan).

### 2.6. Compositional Changes of the Extruded EBC and NBG

#### 2.6.1. Specimens

Specimens retrieved on postoperative Days 1 and 28 (*n* = 6 at each time point: 3 for EBC and 3 for NBG) were examined. The specimens were fixed in a 2.5% glutaraldehyde solution buffered with 60 mmol/L 4-(2-hydroxyethyl)-1-piperazineethanesulfonic acid for 24 h, dehydrated with ethanol and acetone, and embedded into methyl methacrylate (MMA) resin solution (Technovit 9100; Kulzer, Wehrheim, Germany) that was polymerized at −10 °C within 5 days. The resin-embedded specimens were scanned using micro-CT (CosmoScan GX; Rigaku) with the same setting parameters described above. Subsequently, with reference to the micro-CT images, the specimens were ground with a rotary grinding machine (Vector Power Head; BUEHLER, Lake Bluff, IL, USA) until the central part of the extruded sealers was exposed.

#### 2.6.2. EPMA

The extruded sealers on the specimen surfaces were analyzed using EPMA1601 (Shimadzu, Kyoto, Japan). The specimens’ surfaces were polished with diamond pads (Struers, Champigny-sur-Marne, France) and coated with a thin layer of gold using an ion-sputtering device (IC-50; Shimadzu). Elemental mapping of calcium (Ca), phosphorus (P), silicon (Si), zirconium (Zr), and bismuth (Bi) distributions was performed at 400× magnification (568 × 568 μm^2^). The EPMA accelerating voltage was 15 kV, the step size was 4 μm, and the sampling time was 0.1 s at each point. Elemental composition analysis was also conducted with an accelerating voltage of 15 kV and a beam spot size of 10 μm. For each specimen, three different points were measured, and the average value was recorded. The elemental composition on Day 1 was statistically compared with the elemental composition on Day 28 using an unpaired *t*-test, with *p*-values less than 0.05 being considered statistically significant. SPSS 10 was used for statistical analysis.

#### 2.6.3. Micro-Raman Spectrometry

Following EPMA, the specimens underwent micro-Raman spectrometry. The gold coating on the specimens was removed by polishing with diamond pads (Struers), and Raman spectra were collected from the extruded sealers using a micro-Raman spectrometer coupled to a microscope with a 100× objective lens (NRS-3100; JASCO, Tokyo, Japan). The excitation wavelength was 532 nm, and the power was 7.4 mW. Raman spectra of the as-prepared sealers were also measured to detect Raman bands derived from the original components of the sealers.

## 3. Results

### 3.1. Microcomputed Tomography

**Figure 2:**
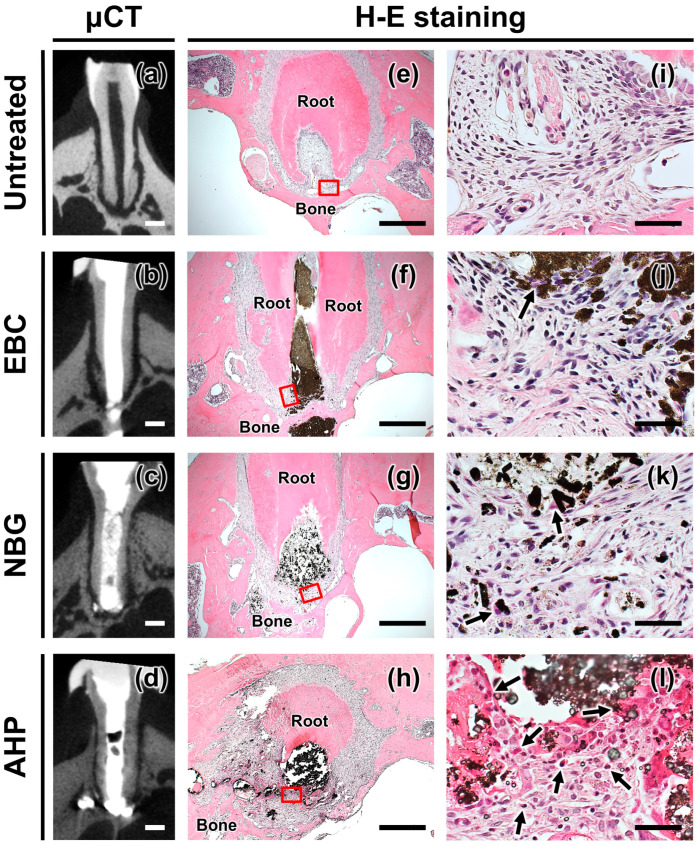
Periapical tissue reactions 28 days after the extrusion of EndoSequence BC Sealer (EBC), Nishika Canal Sealer BG (NBG), and AH Plus (AHP). (**a**–**d**) Representative microcomputed tomography images of untreated teeth and teeth with sealer extrusion showing no periapical lesions (scale: 500 μm). (**e**–**h**) Representative hematoxylin and eosin staining images of untreated teeth and teeth with sealer extrusion. (**i**–**l**) Representation of the boxed areas in (**e**–**h**) at 400× magnification. Only a few inflammatory cells were present around the extruded EBC and NBG, whereas dense inflammatory cell infiltration was observed around the extruded AHP. Arrows indicate inflammatory cells. Scale in (**e**–**h**): 400 μm. Scale in (**i**–**l**): 40 μm.

No periapical lesions were detected in any of the specimens (Figure 2, Table 2). The periapical radiolucency diameter ranged from 0.14 to 0.26 mm in teeth overfilled with EBC, from 0.23 to 0.29 mm in teeth overfilled with NBG, and from 0.24 to 0.35 mm in teeth overfilled with AHP.

### 3.2. Hematoxylin and Eosin Staining

The untreated teeth exhibited the histology of healthy periapical tissues with low densities of scattered macrophages. Neutrophils, lymphocytes, and plasma cells were absent in the tissues (Figure 2). Teeth overfilled with EBC and NBG showed mild or minimal infiltration of inflammatory cells in the periapical tissues (Table 2). Few macrophages were observed, with no neutrophils, lymphocytes, or plasma cells present around the extruded EBC and NBG (Figure 2). The periapical tissues around the extruded AHP showed moderate inflammation with a large number of macrophages. Lymphocytes and plasma cells were also scattered around the extruded AHP. The average numbers of inflammatory cells per field were 1–3.3 for EBC, 0–4.3 for NBG, and 14.3–21.7 for AHP specimens.

### 3.3. Brown–Hopps Staining

**Figure 3:**
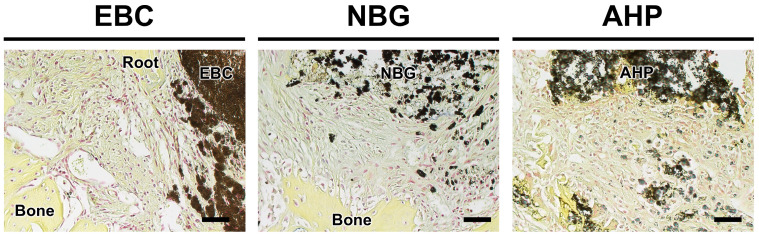
Representative images of periapical tissues stained using Brown–Hopps staining. No positively stained bacteria were observed. EBC: EndoSequence BC Sealer. NBG: Nishika Canal Sealer BG. AHP: AH Plus. Scale: 40 μm.

No bacteria were detected in the periapical tissues using Brown–Hopps staining (Figure 3).

### 3.4. Immunofluorescence

**Figure 4:**
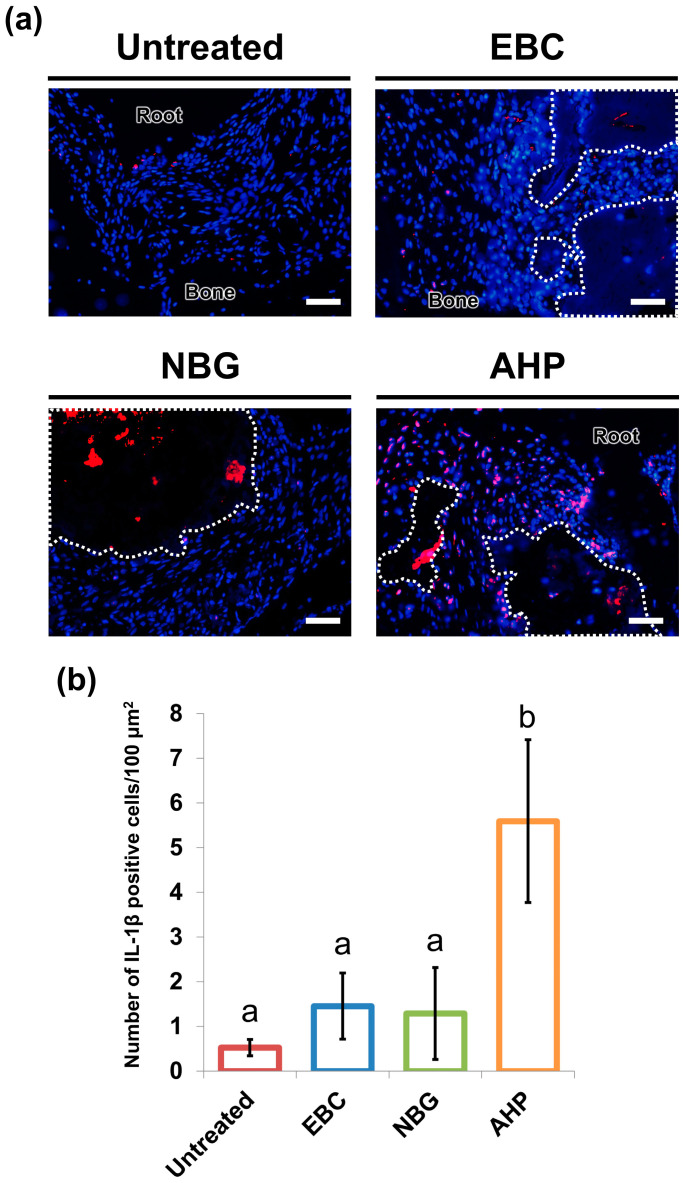
Immunoreactivity of interleukin-1 beta (IL-1β) in the periapical tissues 28 days after the extrusion of EndoSequence BC Sealer (EBC), Nishika Canal Sealer BG (NBG), and AH Plus (AHP). (**a**) Representative images of immunofluorescence staining for IL-1β in untreated teeth and teeth with sealer extrusion. The extruded sealers are outlined using white dotted lines. Red: IL-1β. Blue: DAPI (nuclear stain). Scale: 50 μm. (**b**) Density of IL-1β-positive cells in the periapical tissues. Data are presented as means ± standard deviation. Different letters indicate statistically significant differences (Tukey’s test, n = 6, *p* < 0.05).

Although only a few IL-1β-positive cells were found in normal periapical tissues and around the extruded EBC and NBG, a large number of these cells were found around the extruded AHP. The AHP group exhibited significantly higher IL-1β-positive cell density than the other groups. No significant differences were found in the other comparisons (Figure 4).

### 3.5. EPMA

**Figure 5:**
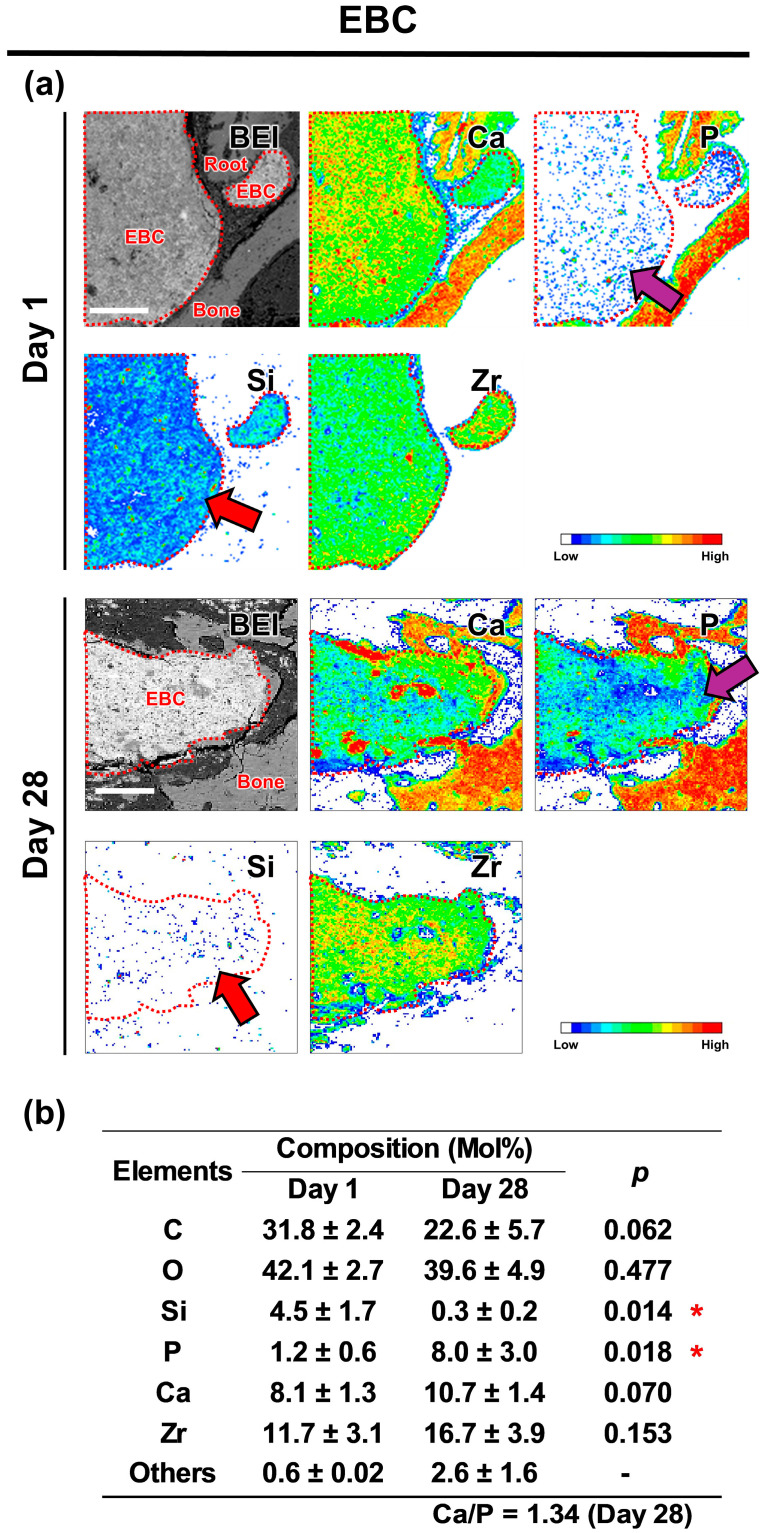
Changes in the elemental composition of EndoSequence BC Sealer (EBC) in periapical tissues. (**a**) Backscattered electron images and elemental mapping images showing the distribution of calcium (Ca), phosphorus (P), silicon (Si), and zirconium (Zr) in the periapical area. EBC is outlined using red dotted lines. On Day 1, moderate intensity Si signals were observed in the extruded EBC, whereas Si signals were hardly detected on Day 28 (red arrows). P signals were scarce in the extruded EBC on Day 1 but showed moderate intensity on Day 28 (purple arrows). Scale: 150 μm. (**b**) Elemental composition of the extruded EBC. Asterisks indicate significant differences in elemental composition between Days 1 and 28 (unpaired *t*-test with *p* < 0.05 indicating statistical significance).

**Figure 6:**
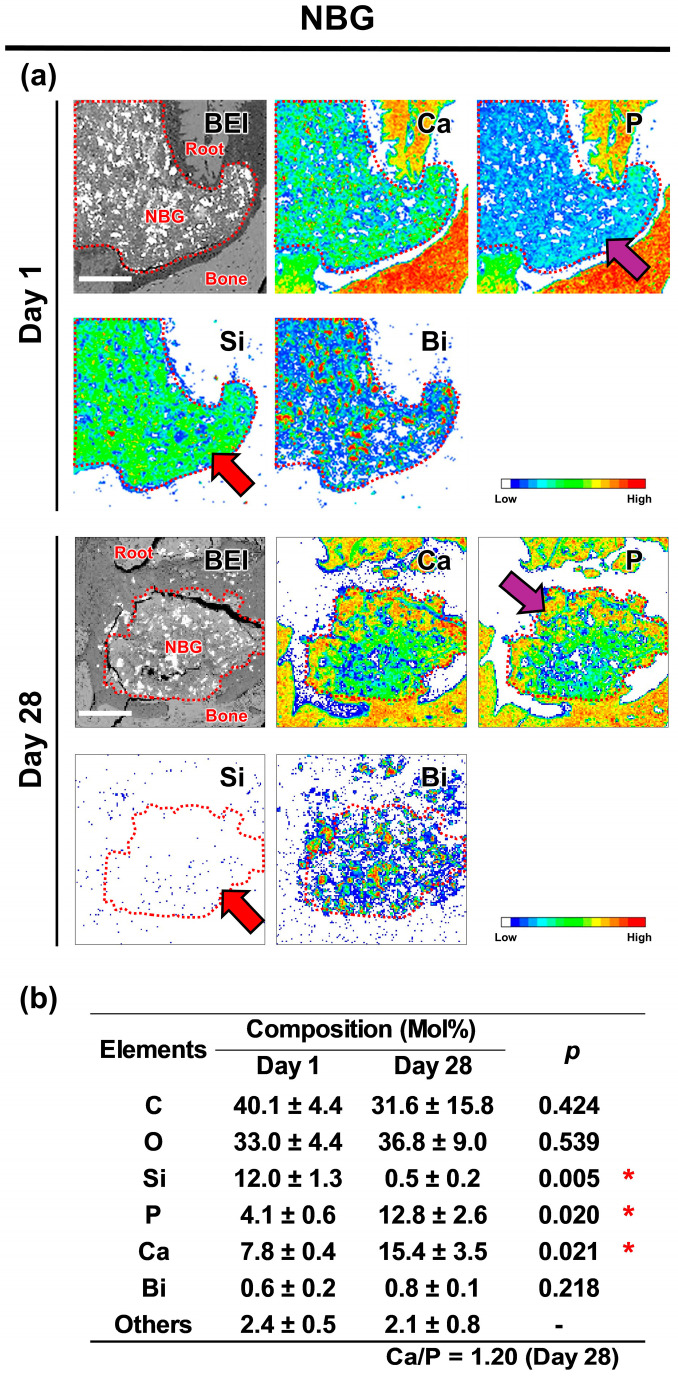
Changes in the elemental composition of Nishika Canal Sealer BG (NBG) in periapical tissues. (**a**) Backscattered electron images and elemental mapping images showing the distribution of calcium (Ca), phosphorus (P), silicon (Si), and bismuth (Bi) in the periapical area. The NBG is outlined using red dotted lines. On Day 1, a moderate intensity of Si signals was observed in the extruded NBG, whereas Si signals were hardly detected on Day 28 (red arrows). P signals were weakly observed in the extruded NBG on Day 1 but showed moderate to high intensity on Day 28 (purple arrows). Scale: 150 μm. (**b**) Elemental composition of the extruded NBG. Asterisks indicate significant differences in elemental composition between Days 1 and 28 (unpaired *t*-test with *p* < 0.05 indicating statistical significance).

The elemental mapping images taken on postoperative Day 1 revealed that the extruded EBC contained Ca, Si, and Zr but only a minimal amount of P (Figure 5), whereas the extruded NBG contained Ca, P, Si, and Bi (Figure 6). By postoperative Day 28, the elemental composition of the extruded EBC and NBG had shifted, now containing Ca, P, and Zr or Bi, with only trace amounts of Si (Figure 5 and Figure 6). The elemental composition analysis indicated that the extruded EBC had a significantly higher P concentration and a significantly lower Si concentration on postoperative Day 28 than on Day 1 (Figure 5), while the extruded NBG had significantly higher concentrations of Ca and P but a significantly lower Si concentration on postoperative Day 28 than on Day 1 (Figure 6).

### 3.6. Raman Spectrometry

**Figure 7:**
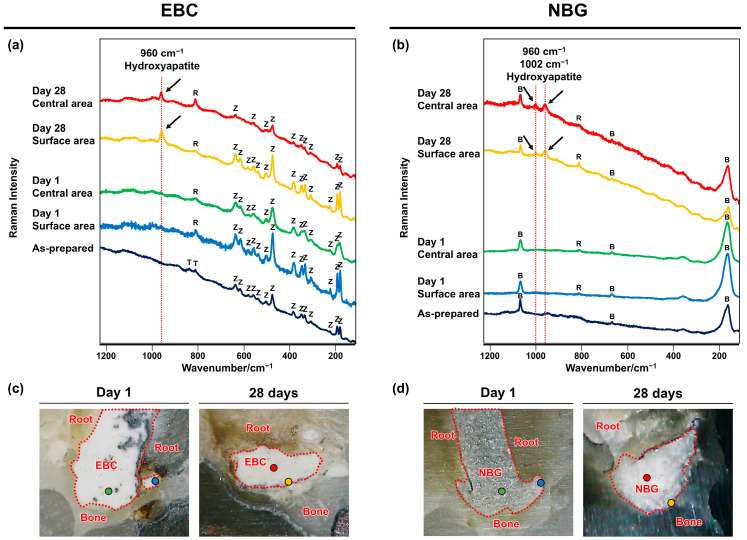
Changes in the chemical composition of EndoSequence BC Sealer (EBC) and Nishika Canal Sealer BG (NBG) in periapical tissues. (**a**,**b**) Raman spectra retrieved from the extruded EBC and NBG and the as-prepared EBC and NBG. The Raman spectra of the as-prepared EBC and NBG showed peaks for zirconium oxide (Z), tricalcium silicate (T), and bismuth subcarbonate (B). Peaks at 812 cm^−1^ were observed in the extruded EBC and NBG due to the embedding resin (R). The extruded EBC and NBG exhibited peaks for hydroxyapatite (arrows) on postoperative Day 28. (**c**,**d**) Bright-field images of the specimens for Raman spectrometry. The EBC and NBG are outlined using red dotted lines. The colored dots in the bright-field images indicate the locations of Raman measurements.

The Raman bands corresponding to zirconium oxide [24], tricalcium silicate [24], and bismuth subcarbonate [25] were identified in the spectra of the as-prepared EBC and NBG. The extruded EBC and NBG in the specimens collected on postoperative Day 1 exhibited only Raman bands attributed to the original components of the sealers and the embedding MMA resin [26] (Figure 7). However, the extruded EBC and NBG in the specimens collected on postoperative Day 28 displayed Raman bands attributed to hydroxyapatite at 960 and 1002 cm^−1^ [27], in addition to Raman bands attributed to their original components and the embedding resin [26] (Figure 7).

## 4. Discussion

Root canal sealers should remain confined to the root canal space. However, sealer extrusion into periapical tissues is not uncommon [28], especially in cases with a wide apical foramen due to apical inflammatory root resorption, incorrect root canal preparation, or pulp necrosis prior to complete root maturation. Previous studies have shown that extrusion of irritating foreign materials into periapical tissues can lead to periapical bone resorption and foreign body granuloma formation, hindering periapical tissue healing [29,30,31]. Therefore, root canal sealers should have low irritancy to periapical tissues and exhibit biocompatibility even in cases of extrusion.

The present study assessed two bioceramic sealers: tricalcium silicate-containing EBC and bioactive glass-containing NBG. Tricalcium silicate-based endodontic cements (mineral trioxide aggregates) have been reported to have osteo-, cemento-, and dentin-inductive properties, promoting tissue repair [32,33,34]. Bioactive glass, used as a bone substitute, has been shown to bond to bone without causing a foreign body reaction [35]. Accordingly, incorporating tricalcium silicate or bioactive glass into root canal sealers shows promise in enhancing their biological properties.

To assess the biocompatibility of EBC and NBG, we used a sealer extrusion model in rat molars. Root canal sealers’ interactions with periapical tissue have mainly been studied in large animal teeth due to technical challenges in performing root canal treatment on small animal teeth [36]. However, recent reports have demonstrated the feasibility of precise root canal treatment on rat molars using a microscope and small instruments [37,38]. Rat models offer advantages due to their periapical anatomy similarity to humans and cost-effectiveness in terms of animal acquisition and breeding.

Periapical tissue reactions were observed 28 days post-overfilling of rat molars. Significant bone resorption and periapical lesion formation occur within 28 days when rat periapical tissues are exposed to strong chemical or bacterial stimuli [39,40].

In the present study, the presence or absence of periapical lesions was assessed using the cone beam CT-periapical index for humans [20]. Despite the size difference between rat and human teeth, the normal periodontal ligament thickness is comparable in both species [41,42]. Therefore, we deemed it appropriate to apply the human periapical index to rats.

The severity of periapical inflammation caused by sealer extrusion was evaluated by quantifying the number of inflammatory cells and IL-1β-immunopositive cells around the extruded sealers. IL-1β is a proinflammatory cytokine produced by various types of cells, including macrophages, neutrophils, fibroblasts, osteoblasts, and endothelial cells [43]. The accumulation of IL-1β-immunopositive cells has been observed in inflamed tissues, such as periapical lesions [44] and inflamed pulp [45]. In fact, a positive correlation has been identified between the level of IL-1β synthesis and the severity of inflammation in periapical lesions [46,47].

The micro-CT analysis in this study revealed that the extrusion of EBC and NBG did not lead to the formation of periapical lesions. The hematoxylin and eosin staining demonstrated that the intensity of inflammatory cell infiltration around the extruded EBC and NBG ranged from absent to mild at 28 days. The immunofluorescence staining further indicated that IL-1β-positive cell density in periapical tissues did not significantly differ between untreated teeth and teeth overfilled with EBC and NBG. These findings indicate that the extruded EBC and NBG had favorable biocompatibility and did not cause considerable damage to the periapical tissues of rats. Therefore, the first null hypothesis was rejected.

The present findings are consistent with previous findings demonstrating favorable responses of periapical cells and tissues to EBC and NBG. In vitro studies have shown that EBC and NBG elutes do not adversely affect cell viability or migration of periodontal ligament stem cells and osteoblasts [12,48,49,50]. A case report on accidental EBC extrusion also reported no inflammatory cells or foreign body giant cells around the extruded EBC 7 weeks after sealer extrusion [17]. Additionally, an animal study revealed that NBG root canal filling did not impede the healing of periapical tissues in rat molars [51]. Collectively, these findings support the safety of EBC and NBG for clinical use.

EBC and NBG are commonly used for single-cone root canal filling. This technique requires less effort and time compared to conventional cold lateral and warm vertical obturation methods [52]. However, the use of a relatively large volume of sealer in single-cone fillings may increase the risk of sealer extrusion into the periapical tissues [53]. Although previous studies have shown that extrusion of EBC did not significantly decrease the long-term success rate of endodontic treatment [54,55], its potential to cause delayed healing remains unclear. Furthermore, the impact of NBG extrusion on endodontic treatment outcomes has yet to be reported. In our study, both the extruded EBC and NBG exhibited minimal irritancy to periapical tissues. These findings suggest that the extrusion of EBC and NBG is unlikely to delay or disrupt periapical tissue healing, thereby supporting the efficacy of the single-cone method using these sealers.

Our findings suggest that EBC and NBG extrusion do not result in persistent inflammation in periapical tissues. However, EBC extrusion may lead to increased early postoperative pain due to its high alkalinity [56]. Furthermore, extrusion of any sealer can induce facial dysesthesia [57] and maxillary sinusitis [58] if they reach the inferior alveolar canal or maxillary sinus. Therefore, regardless of the irritancy of the sealers, care should be taken to prevent sealer extrusion.

The present study employed an epoxy resin-based AHP sealer as a reference. While this sealer exhibits very low solubility and excellent sealing properties [2], its biological characteristics remain suboptimal. Previous research has reported that AHP can release cytotoxic components, such as bisphenol A diglycidyl ether and 1-adamantylamine, even after hardening [59]. Additionally, eluates from AHP have been shown to increase the production of inflammatory cytokines, including IL-6, IL-8, and growth-regulated oncogene [60], in periodontal ligament cells. Clinical studies have also suggested that the extrusion of AHP into periapical tissues may delay the healing process following root canal treatment [61,62]. Consequently, root canal sealers that exhibit lower cytotoxicity and reduced irritancy to periapical tissues compared to AHP are in clinical demand.

In the present study, moderate inflammatory cell infiltration was observed around the extruded AHP. Considering that no bacterial infection was detected through Brown–Hopps staining, this tissue reaction can be attributed to the tissue irritancy of AHP. Conversely, the inflammation around the extruded EBC and NBG had nearly resolved. Furthermore, significantly less IL-1β-positive cells were observed around the extruded EBC and NBG than around the extruded AHP. These findings suggest that EBC and NBG cause less irritation to periapical tissues than AHP. However, it is important to note that the amount of sealer extrusion was not standardized in the current study. Since the periapical tissue response to sealer extrusion depends on the properties of the material and the quantity extruded, further research using well-standardized in vivo experimental models is required to comprehensively evaluate the periapical tissue irritancy of various sealers in comparison to AHP.

The present study also investigated the compositional changes of the extruded EBC and NBG. Previous research has shown that EBC and NBG attract Ca and P ions in artificial body fluids, leading to the production of hydroxyapatite on the surface [12,24]. Moreover, our previous studies demonstrated that EBC formed hydroxyapatite on the surface when implanted in the subcutaneous tissue of rats, while NBG formed a silicon-depleted area within the material [63,64]. However, the present study is the first report on the chemical behavior of EBC and NBG in periapical tissues.

The results of the present EPMA and micro-Raman analysis showed significant changes in the composition of the extruded EBC and NBG from Day 1 to 28, rejecting the second null hypothesis of the present study. Specifically, the concentration of Si elements in the extruded EBC and NBG decreased significantly over time, with complete depletion by Day 28. This suggests that the silica components, namely calcium silicate hydrate and bioactive glass, in the extruded sealers were dissolved, releasing a large amount of Si elements into the surrounding tissues. Furthermore, on Day 28, the extruded EBC and NBG exhibited increased levels of Ca and P, along with Raman peaks indicative of hydroxyapatite formation on both the surface and central areas. These results indicate that the extruded sealers absorbed Ca and P from the surrounding environment, leading to hydroxyapatite formation throughout the material.

The mechanism underlying these compositional changes in the extruded EBC and NBG remains unclear. However, hardened EBC and NBG contain calcium silicate hydrate and bioactive glass as primary components. Furthermore, it is known that, when these components interact with physiological solutions, they dissolve to form a silica gel layer that promotes hydroxyapatite formation [65]. Therefore, the observed changes in composition may be attributed to the penetration of body fluids into the center of the extruded EBC and NBG, leading to the dissolution of calcium silicate hydrate or bioactive glass, which subsequently induced the formation of hydroxyapatite.

The release of Si elements and the formation of hydroxyapatite in the extruded EBC and NBG may have had a biological effect on the surrounding tissues. Previous studies have shown that a silicate ion-rich extract from calcium silicate-based biomaterials can attenuate inflammation in the aorta and lungs of mice [66,67]. Therefore, the Si elements released from the extruded EBC and NBG may have contributed to the early resolution of inflammation in periapical tissues by increasing the concentration of silicate ions. Meanwhile, hydroxyapatite, similar to the mineral component of bones and teeth, is known for its high biocompatibility [68,69]. Furthermore, nanosized hydroxyapatite has been shown to promote the polarization of macrophages into the M2 phenotype [70,71], which secretes anti-inflammatory and tissue repair mediators including IL-10, IL-1 receptor antagonist, and transforming growth factor beta [72]. Therefore, the formation of hydroxyapatite in the extruded EBC and NBG may have enhanced the biocompatibility of these materials.

Notably, the Ca/P ratios of the extruded EBC and NBG measured on postoperative Day 28 (1.34 and 1.20, respectively) were lower than the stoichiometric Ca/P ratio of hy-droxyapatite (1.67) [73]. This suggests that the extruded EBC and NBG contain Ca-deficient hydroxyapatite (Ca/P: 1.5–1.67) or Ca-poor apatite precursors, such as dicalcium phosphate (Ca/P: 1), octacalcium phosphate (Ca/P: 1.3), or amorphous calcium phosphate (Ca/P: 1.5) [73]. Crystallized hydroxyapatite is stable and exhibits low solubility in vivo [74]. However, calcium phosphate phases with lower Ca/P ratios and reduced crystallinity, such as those potentially present in the extruded EBC and NBG, demonstrate higher solubility [73]. Thus, it can be inferred that the extruded EBC and NBG have a certain degree of solubility, gradually dissolving and eventually disappearing from the periapical tissues after an extended period.

The present study, conducted in rat molars, has some limitations. Due to the smaller size of rat molars compared to human teeth, the amount of extruded sealers observed in the present study was considerably lower than what is typically seen in clinical practice. Different physicochemical behaviors may be exhibited by small and large masses of extruded sealers in periapical tissues, potentially affecting the surrounding tissues differently. Furthermore, rats share a similar immune response to foreign materials as humans; however, it is not exactly identical [75]. Consequently, the findings of this study may not be directly applicable to clinical settings. However, collecting clinical samples of sealer extrusion, particularly those without infection symptoms, presents ethical challenges. Despite these limitations, the in vivo animal experimental data on sealer extrusion remain valuable for advancing our understanding of these phenomena.

Another limitation of the present study is that the biocompatibility of EBC and NBG was only evaluated when these materials were extruded into normal periapical tissues without bone loss. In clinical practice, sealers are sometimes extruded into bone defects in periapical lesions, potentially impairing the bone repair process due to the irritating effect of the extruded sealers [62,76]. Therefore, future research should assess the impact of EBC and NBG extrusion on the repair of periapical bone defects using teeth with periapical lesions.

In addition to EBC and NBG, a wide range of bioceramic sealers are commercially available. These are generally characterized by low cytotoxicity and offer several distinct advantages [77]. For example, AH Plus Bioceramic Sealer (Dentsply Sirona) has a notably rapid setting time and low solubility [78], while the viscosity of EndoSequence BC Sealer HiFlow (Brasseler) decreases when heated, making it particularly suitable for warm vertical obturation techniques [79]. However, the safety of these bioceramic sealers, particularly when extruded beyond the apical foramen, requires comprehensive evaluation prior to widespread clinical use. Our rat model is a potentially valuable tool for future studies focused on assessing the biocompatibility of bioceramic sealers.

## 5. Conclusions

EBC and NBG demonstrated favorable biocompatibility, with the potential to release silicon elements and generate hydroxyapatite when extruded into the periapical tissues of rats. These findings enhance our understanding of the biological and chemical characteristics of EBC and NBG, suggesting that they are viable options as root canal sealers.

## Figures and Tables

**Table 1:** Compositions of the sealers used in the present study.

Materials	Lot No.	Composition
EndoSequence BC Sealer (EBC)	23001SP	Zirconium oxide, tricalcium silicate, dicalcium silicate, calcium hydroxide, calcium phosphate monobasic, filler, and thickening agents
Nishika Canal Sealer BG (NBG)	M7P	*Paste A:* fatty acid, bismuth subcarbonate, and silicon dioxide *Paste B:* magnesium oxide, calcium silicate glass (bioactive glass), and silicon dioxide
AH Plus (AHP)	2204000799	*Paste A:* bisphenol-A epoxy resin, bisphenol-F epoxy resin, calcium tungstate, zirconium oxide, silica, and pigment *Paste B:* N-dibenziyl-5-oxanonane, aminoadamantane, tricyclodecane-diamine, calcium tungstate, zirconium oxide, silica, and silicone oil

**Table 2:** Summary of periapical tissue reactions to sealer extrusion.

Time	Material	Periapical Lesions		Inflammatory Infiltrates			
		Absent	Present	Absent/ Very Few	Mild	Moderate	Severe
Day 28	EndoSequence BC Sealer (EBC)	6	0	5	1	0	0
	Nishika Canal Sealer BG (NBG)	6	0	4	2	0	0
	AH Plus (AHP)	6	0	0	0	6	0

## Data Availability

The original contributions presented in the study are included in the article, further inquiries can be directed to the corresponding author.

## References

[1] Evans J.T., Simon J.H. (1986). Evaluation of the apical seal produced by injected thermoplasticized gutta-percha in the absence of smear layer and root canal sealer. J. Endod..

[2] Komabayashi T., Colmenar D., Cvach N., Bhat A., Primus C., Imai Y. (2020). Comprehensive review of current endodontic sealers. Dent. Mater. J..

[3] Slaus G., Bottenberg P. (2002). A survey of endodontic practice amongst Flemish dentists. Int. Endod. J..

[4] Guo J., Peters O.A., Hosseinpour S. (2023). Immunomodulatory Effects of Endodontic Sealers: A Systematic Review. Dent. J..

[5] Donnermeyer D., Bürklein S., Dammaschke T., Schäfer E. (2018). Endodontic sealers based on calcium silicates: A systematic review. Odontology.

[6] Zhou H.-M., Shen Y., Zheng W., Li L., Zheng Y.F., Haapasalo M. (2013). Physical properties of 5 root canal sealers. J. Endod..

[7] Kwak S.W., Koo J., Song M., Jang I.H., Gambarini G., Kim H.-C. (2023). Physicochemical Properties and Biocompatibility of Various Bioceramic Root Canal Sealers: In Vitro Study. J. Endod..

[8] Zhang W., Li Z., Peng B. (2009). Assessment of a new root canal sealer’s apical sealing ability. Oral Surg. Oral Med. Oral Pathol. Oral Radiol. Endodontol..

[9] Rodríguez-Lozano F.J., López-García S., García-Bernal D., Tomás-Catalá C.J., Santos J.M., Llena C., Lozano A., Murcia L., Forner L. (2020). Chemical composition and bioactivity potential of the new Endosequence BC Sealer formulation HiFlow. Int. Endod. J..

[10] Giacomino C.M., Wealleans J.A., Kuhn N., Diogenes A. (2019). Comparative Biocompatibility and Osteogenic Potential of Two Bioceramic Sealers. J. Endod..

[11] Yoshii S., Washio A., Morotomi T., Kitamura C. (2016). Root canal sealing ability of bioactive glass-based sealer and its effects on dentin. Jpn. J. Conserv. Dent..

[12] Bin Jo S., Kim H.K., Lee H.N., Kim Y.-J., Patel K.D., Knowles J.C., Lee J.-H., Song M. (2020). Physical Properties and Biofunctionalities of Bioactive Root Canal Sealers In Vitro. Nanomaterials.

[13] Vert M., Doi Y., Hellwich K.-H., Hess M., Hodge P., Kubisa P., Rinaudo M., Schué F. (2012). Terminology for biorelated polymers and applications (IUPAC recommendations 2012). Pure Appl. Chem..

[14] Silva E., Tanomaru-Filho M., Silva G., Lopes C., Cerri P., Tanomaru J.G. (2021). Evaluation of the biological properties of two experimental calcium silicate sealers: An in vivo study in rats. Int. Endod. J..

[15] Kato A., Miyaji H., Yoshino Y., Kanemoto Y., Hamamoto A., Nishida E., Sugaya T., Tanaka S. (2022). In Vivo Inflammatory Effects and Surface Composition Changes in Implanted Root Canal Sealer Containing Bioactive Glass. Jpn. J. Conserv. Dent..

[16] Almeida L.H., Gomes A.P.N., Gastmann A.H., Pola N.M., Moraes R.R., Morgental R.D., Cava S.S., Felix A.O.C., Pappen F.G., Almeida L.H. (2019). Bone tissue response to an MTA-based endodontic sealer, and the effect of the addition of calcium aluminate and silver particles. Int. Endod. J..

[17] Ricucci D., Grande N.M., Plotino G., Tay F.R. (2020). Histologic Response of Human Pulp and Periapical Tissues to Tricalcium Silicate–based Materials: A Series of Successfully Treated Cases. J. Endod..

[18] Ikarashi A., Sano H., Tanaka M., Ohshima H. (2023). The accuracy of quantifying the degree of hard tissue calcification using an electron probe micro analyzer, micro-focus X-ray computed tomography, and tissue sectioning methods. J. Oral Biosci..

[19] Kazanci M., Fratzl P., Klaushofer K., Paschalis E.P. (2006). Complementary information on in vitro conversion of amorphous (precursor) calcium phosphate to hydroxyapatite from Raman microspectroscopy and wide-angle X-ray scattering. Calcif. Tissue Int..

[20] Estrela C., Bueno M.R., Azevedo B.C., Azevedo J.R., Pécora J.D. (2008). A new periapical index based on cone beam computed tomography. J. Endod..

[21] da Fonseca T.S., da Silva G.F., Tanomaru-Filho M., Sasso-Cerri E., Guerreiro-Tanomaru J.M., Cerri P.S. (2015). In vivo evaluation of the inflammatory response and IL-6 immunoexpression promoted by Biodentine and MTA Angelus. Int. Endod. J..

[22] Holland R., Júnior A.S., de Souza V., Junior E.D., Filho J.A.O., Bernabé P.F.E., Nery M.J., Murata S.S. (2005). Influence of apical patency and filling material on healing process of dogs’ teeth with vital pulp after root canal therapy. Braz. Dent. J..

[23] Edanami N., Yoshiba N., Ohkura N., Takeuchi R., Tohma A., Noiri Y., Yoshiba K. (2017). Characterization of Dental Pulp Myofibroblasts in Rat Molars after Pulpotomy. J. Endod..

[24] Zamparini F., Siboni F., Prati C., Taddei P., Gandolfi M.G. (2018). Properties of calcium silicate-monobasic calcium phosphate materials for endodontics containing tantalum pentoxide and zirconium oxide. Clin. Oral Investig..

[25] Zapata G.E.T., Etcheverry S.B., Baran E.J. (1997). Vibrational spectrum of bismuth subcarbonate. J. Mater. Sci. Lett..

[26] Matamoros-Ambrocio M., Sánchez-Mora E., Gómez-Barojas E., Luna-López J.A. (2021). Synthesis and Study of the Optical Properties of PMMA Microspheres and Opals. Polymers.

[27] Gandolfi M.G., Taddei P., Tinti A., Prati C. (2010). Apatite-forming ability (bioactivity) of ProRoot MTA. Int. Endod. J..

[28] Ng Y.-L., Mann V., Gulabivala K. (2011). A prospective study of the factors affecting outcomes of nonsurgical root canal treatment: Part 1: Periapical health. Int. Endod. J..

[29] Nair P.R., Sjögren U., Krey G., Sundqvist G. (1990). Therapy-resistant foreign body giant cell granuloma at the periapex of a root-filled human tooth. J. Endod..

[30] Ricucci D., Siqueira J.F., Bate A.L., Ford T.R.P. (2009). Histologic investigation of root canal–treated teeth with apical periodontitis: A retrospective study from twenty-four patients. J. Endod..

[31] Fristad I., Molven O., Halse A. (2004). Nonsurgically retreated root filled teeth—Radiographic findings after 20–27 years. Int. Endod. J..

[32] Gandolfi M., Iezzi G., Piattelli A., Prati C., Scarano A. (2017). Osteoinductive potential and bone-bonding ability of ProRoot MTA, MTA Plus and Biodentine in rabbit intramedullary model: Microchemical characterization and histological analysis. Dent. Mater..

[33] Baek S.-H., Plenk H., Kim S. (2005). Periapical tissue responses and cementum regeneration with Amalgam, SuperEBA, and MTA as root-end filling materials. J. Endod..

[34] Edanami N., Ibn Belal R.S., Yoshiba K., Yoshiba N., Ohkura N., Takenaka S., Noiri Y. (2021). Effect of a resin-modified calcium silicate cement on inflammatory cell infiltration and reparative dentin formation after pulpotomy in rat molars. Aust. Endod. J..

[35] Skallevold H.E., Rokaya D., Khurshid Z., Zafar M.S. (2019). Bioactive Glass Applications in Dentistry. Int. J. Mol. Sci..

[36] Bernáth M., Szabó J. (2003). Tissue reaction initiated by different sealers. Int. Endod. J..

[37] Yoneda N., Noiri Y., Matsui S., Kuremoto K., Maezono H., Ishimoto T., Nakano T., Ebisu S., Hayashi M. (2017). Development of a root canal treatment model in the rat. Sci. Rep..

[38] Edanami N., Yoshiba K., Shirakashi M., Ibn Belal R.S., Yoshiba N., Ohkura N., Tohma A., Takeuchi R., Okiji T., Noiri Y. (2020). Impact of remnant healthy pulp and apical tissue on outcomes after simulated regenerative endodontic procedure in rat molars. Sci. Rep..

[39] Erausquin J., Muruzábal M. (1967). Root canal fillings with zinc oxide-eugenol cement in the rat molar. Oral Surg. Oral Med. Oral Pathol..

[40] Xiong B., Shirai K., Matsumoto K., Abiko Y., Furuichi Y. (2020). The potential of a surface pre-reacted glass root canal dressing for treating apical periodontitis in rats. Int. Endod. J..

[41] Cuoghi O.A., Tondelli P.M., Aiello C.A., de Mendonça M.R., da Costa S.C. (2013). Importance of periodontal ligament thickness. Braz. Oral Res..

[42] Nanci A., Bosshardt D.D. (2006). Structure of periodontal tissues in health and disease. Periodontology 2000.

[43] Tazawa K., Presse M.M.A., Furusho H., Stashenko P., Sasaki H. (2022). Revisiting the role of IL-1 signaling in the development of apical periodontitis. Front. Dent. Med..

[44] Matsumoto A., Anan H., Maeda K. (1998). An immunohistochemical study of the behavior of cells expressing interleukin-1 alpha and interleukin-1 beta within experimentally induced periapical lesions in rats. J. Endod..

[45] Silva A.C.O., Faria M.R., Fontes A., Campos M.S., Cavalcanti B.N. (2009). Interleukin-1 beta and interleukin-8 in healthy and inflamed dental pulps. J. Appl. Oral Sci..

[46] Yang N.-Y., Zhou Y., Zhao H.-Y., Liu X.-Y., Sun Z., Shang J.-J. (2018). Increased interleukin 1α and interleukin 1β expression is involved in the progression of periapical lesions in primary teeth. BMC Oral Health.

[47] Jakovljevic A., Knezevic A., Karalic D., Soldatovic I., Popovic B., Milasin J., Andric M. (2014). Pro-inflammatory cytokine levels in human apical periodontitis: Correlation with clinical and histological findings. Aust. Endod. J..

[48] Washio A., Nakagawa A., Nishihara T., Maeda H., Kitamura C. (2014). Physicochemical properties of newly developed bioactive glass cement and its effects on various cells. J. Biomed. Mater. Res. Part B Appl. Biomater..

[49] López-García S., Myong-Hyun B., Lozano A., García-Bernal D., Forner L., Llena C., Guerrero-Gironés J., Murcia L., Rodríguez-Lozano F.J. (2019). Cytocompatibility, bioactivity potential, and ion release of three premixed calcium silicate-based sealers. Clin. Oral Investig..

[50] Zhang W., Li Z., Peng B. (2010). Effects of iRoot SP on Mineralization-related Genes Expression in MG63 Cells. J. Endod..

[51] Morotomi T., Hanada K., Washio A., Yoshii S., Matsuo K., Kitamura C. (2017). Effect of newly-developed bioactive glass root canal sealer on periapical tissue of rat’s molar. Jpn. J. Conserv. Dent..

[52] Kim S.R., Kwak S.W., Lee J., Goo H., Ha J., Kim H. (2019). Efficacy and retrievability of root canal filling using calcium silicate-based and epoxy resin-based root canal sealers with matched obturation techniques. Aust. Endod. J..

[53] Fonseca B., Coelho M.S., Bueno C.E.d.S., Fontana C.E., De Martin A.S., Rocha D.G.P. (2019). Assessment of Extrusion and Postoperative Pain of a Bioceramic and Resin-Based Root Canal Sealer. Eur. J. Dent..

[54] Li J., Chen L., Zeng C., Liu Y., Gong Q., Jiang H. (2022). Clinical outcome of bioceramic sealer iRoot SP extrusion in root canal treatment: A retrospective analysis. Head Face Med..

[55] Chybowski E.A., Glickman G.N., Patel Y., Fleury A., Solomon E., He J. (2018). Clinical Outcome of Non-Surgical Root Canal Treatment Using a Single-cone Technique with Endosequence Bioceramic Sealer: A Retrospective Analysis. J. Endod..

[56] Yu Y.-H., Kushnir L., Kohli M., Karabucak B. (2021). Comparing the incidence of postoperative pain after root canal filling with warm vertical obturation with resin-based sealer and sealer-based obturation with calcium silicate-based sealer: A prospective clinical trial. Clin. Oral Investig..

[57] Stanley E., Strother K.K., Kirkpatrick T., Jeong J.W. (2023). Calcium Silicate–based Sealer Extrusion into the Mandibular Canal: 3 Different Recovery Outcomes—A Report of 3 Cases. J. Endod..

[58] Lin J., Wang C., Wang X., Chen F., Zhang W., Sun H., Yan F., Pan Y., Zhu D., Yang Q. (2024). Expert consensus on odontogenic maxillary sinusitis multi-disciplinary treatment. Int. J. Oral Sci..

[59] Graunaite I., Lodiene G., Arandarcikaite O., Pukalskas A., Machiulskiene V. (2018). Leachables and cytotoxicity of root canal sealers. J. Oral Sci..

[60] Gaudin A., Tolar M., Peters O.A. (2020). Cytokine Production and Cytotoxicity of Calcium Silicate–based Sealers in 2- and 3-dimensional Cell Culture Models. J. Endod..

[61] Martins J.B., Scheeren B., van der Waal S. (2023). The Effect of Unintentional AH-Plus Sealer Extrusion on Resolution of Apical Periodontitis After Root Canal Treatment and Retreatment—A Retrospective Case-control Study. J. Endod..

[62] Sari Ş., Durutűrk L. (2007). Radiographic evaluation of periapical healing of permanent teeth with periapical lesions after extrusion of AH Plus sealer. Oral Surg. Oral Med. Oral Pathol. Oral Radiol. Endodontol..

[63] Edanami N., Takenaka S., Ibn Belal R.S., Yoshiba K., Takahara S., Yoshiba N., Ohkura N., Noiri Y. (2023). In Vivo Assessment of the Apatite-Forming Ability of New-Generation Hydraulic Calcium Silicate Cements Using a Rat Subcutaneous Implantation Model. J. Funct. Biomater..

[64] Ibn Belal R.S., Edanami N., Yoshiba K., Yoshiba N., Ohkura N., Takenaka S., Noiri Y. (2021). Comparison of calcium and hydroxyl ion release ability and in vivo apatite-forming ability of three bioceramic-containing root canal sealers. Clin. Oral Investig..

[65] Niu L.-N., Jiao K., Wang T.-D., Zhang W., Camilleri J., Bergeron B.E., Feng H.-L., Mao J., Chen J.-H., Pashley D.H. (2014). A review of the bioactivity of hydraulic calcium silicate cements. J. Dent..

[66] Que Y., Zhang Z., Zhang Y., Li X., Chen L., Chen P., Ou C., Yang C., Chang J. (2022). Silicate ions as soluble form of bioactive ceramics alleviate aortic aneurysm and dissection. Bioact. Mater..

[67] Chen T., Zhang Z., Weng D., Lu L., Wang X., Xing M., Qiu H., Zhao M., Shen L., Zhou Y. (2021). Ion therapy of pulmonary fibrosis by inhalation of ionic solution derived from silicate bioceramics. Bioact. Mater..

[68] Takata T., Katauchi K., Akagawa Y., Nikai H. (1993). New periodontal ligament formation on a synthetic hydroxyapatite surface. Clin. Oral Implant. Res..

[69] Oshima M., Inoue K., Nakajima K., Tachikawa T., Yamazaki H., Isobe T., Sugawara A., Ogawa M., Tanaka C., Saito M. (2014). Functional tooth restoration by next-generation bio-hybrid implant as a bio-hybrid artificial organ replacement therapy. Sci. Rep..

[70] Mahon O.R., Browe D.C., Gonzalez-Fernandez T., Pitacco P., Whelan I.T., Von Euw S., Hobbs C., Nicolosi V., Cunningham K.T., Mills K.H. (2020). Nano-particle mediated M2 macrophage polarization enhances bone formation and MSC osteogenesis in an IL-10 dependent manner. Biomaterials.

[71] Linares J., Fernández A.B., Feito M.J., Matesanz M.C., Sánchez-Salcedo S., Arcos D., Vallet-Regí M., Rojo J.M., Portolés M.T. (2016). Effects of nanocrystalline hydroxyapatites on macrophage polarization. J. Mater. Chem. B.

[72] Rőszer T. (2015). Understanding the Mysterious M2 Macrophage through Activation Markers and Effector Mechanisms. Mediat. Inflamm..

[73] Sheikh Z., Abdallah M.-N., Hanafi A.A., Misbahuddin S., Rashid H., Glogauer M. (2015). Mechanisms of in Vivo Degradation and Resorption of Calcium Phosphate Based Biomaterials. Materials.

[74] Wach T., Kozakiewicz M. (2020). Fast-Versus Slow-Resorbable Calcium Phosphate Bone Substitute Materials—Texture Analysis after 12 Months of Observation. Materials.

[75] Holsapple M.P., West L.J., Landreth K.S. (2003). Species comparison of anatomical and functional immune system development. Birth Defects Res. Part B Dev. Reprod. Toxicol..

[76] Zmener O., Banegas G., Pameijer C.H. (2005). Bone tissue response to a methacrylate-based endodontic sealer: A histological and histometric study. J. Endod..

[77] Eren S.K. (2023). Clinical applications of calcium silicate-based materials: A narrative review. Aust. Dent. J..

[78] Zamparini F., Prati C., Taddei P., Spinelli A., Di Foggia M., Gandolfi M.G. (2022). Chemical-Physical Properties and Bioactivity of New Premixed Calcium Silicate-Bioceramic Root Canal Sealers. Int. J. Mol. Sci..

[79] Ashkar I., Sanz J.L., Forner L., Ghilotti J., Melo M. (2024). A Literature Review of the Effect of Heat on the Physical-Chemical Properties of Calcium Silicate–Based Sealers. J. Endod..

